# Washed microbiota transplantation stopped the deterioration of amyotrophic lateral sclerosis: The first case report and narrative review

**DOI:** 10.7555/JBR.36.20220088

**Published:** 2022-06-28

**Authors:** Gaochen Lu, Quan Wen, Bota Cui, Qianqian Li, Faming Zhang

**Affiliations:** 1 Medical Center for Digestive Diseases, the Second Affiliated Hospital of Nanjing Medical University, Nanjing, Jiangsu 210011, China; 2 Key Lab of Holistic Integrative Enterology, Nanjing Medical University, Nanjing, Jiangsu 210011, China; 3 Department of Microbiotherapy, Sir Run Run Hospital, Nanjing Medical University, Nanjing, Jiangsu 211166, China; 4 National Clinical Research Center for Digestive Diseases, Xi'an, Shanxi 710032, China

**Keywords:** fecal microbiota transplant, microbiome, efficacy, neurodegeneration, amyotrophic lateral sclerosis

## Abstract

Amyotrophic lateral sclerosis (ALS) is known as a progressive paralysis disorder characterized by degeneration of upper and lower motor neurons, and has an average survival time of three to five years. Growing evidence has suggested a bidirectional link between gut microbiota and neurodegeneration. Here we aimed to report one female case with ALS, who benefited from washed microbiota transplantation (WMT), an improved fecal microbiota transplantation (FMT), through a transendoscopic enteral tube during a 12-month follow-up. Notedly, the accidental scalp trauma the patient suffered later was treated with prescribed antibiotics that caused ALS deterioration. The subsequent rescue WMTs successfully stopped the progression of the disease with a quick improvement. The plateaus and reversals occurred during the whole course of WMT. The stool and blood samples from the first WMT to the last were collected for dynamic microbial and metabolomic analysis. We observed the microbial and metabolomic changing trend consistent with the disease status. This case report for the first time shows the direct clinical evidence on using WMT for treating ALS, indicating that WMT may be the novel treatment strategy for controlling this so-called incurable disease.

## Introduction

Amyotrophic lateral sclerosis (ALS) is a systemic disorder that involves dysfunction of multiple organs. There is a large spectrum of clinical presentations in patients with ALS. The most common ones are muscle weakness and impairment in mobility of the limbs^[1]^. In 25% of the affected cases, the bulbar-onset ALS occurs with oropharyngeal muscle involvement, therefore affecting the function of swallowing and speech^[1]^. Besides motor impairment, some patients may develop cognitive or behavioral impairments due to the degeneration in the frontal and temporal lobes.

Presently only riluzole^[2]^ and edaravone^[3]^ have shown some efficacy to some extent, though extensive efforts are being made to develop ALS-specific drugs. In recent clinical trials, several pharmacological treatments, such as the calcium sensitizer levosimendan^[4]^ and the combination of sodium phenylbutyrate and taurursodiol^[5]^ (known as PB-TURSO), have been investigated. However, the results were not satisfactory, and there is still a long way for these treatments to be approved for legitimate use in humans.

Recent studies demonstrated that gut dysbiosis may be a potential factor contributing to the development of ALS in both mice and humans^[6–8]^. These studies indicated the potential to use microbial biomarkers for ALS diagnosis and to manipulate microbiome for the treatment. It has also been reported that gastrointestinal motor dysfunction can occur in ALS, manifested as delayed gastric emptying and colonic transit times. Here we aimed to report the first human case with ALS accompanied by refractory constipation, who benefited from the washed microbiota transplantation (WMT) during a 12-month follow-up, and discussed this topic with a narrative review.

The study was approved by the Research Ethics Committee of the Second Affiliated Hospital of Nanjing Medical University ([2012] KY No. 026), China. Informed consent was obtained for the publication of the clinical photographs from this patient.

## Case report

**Figure 1:**
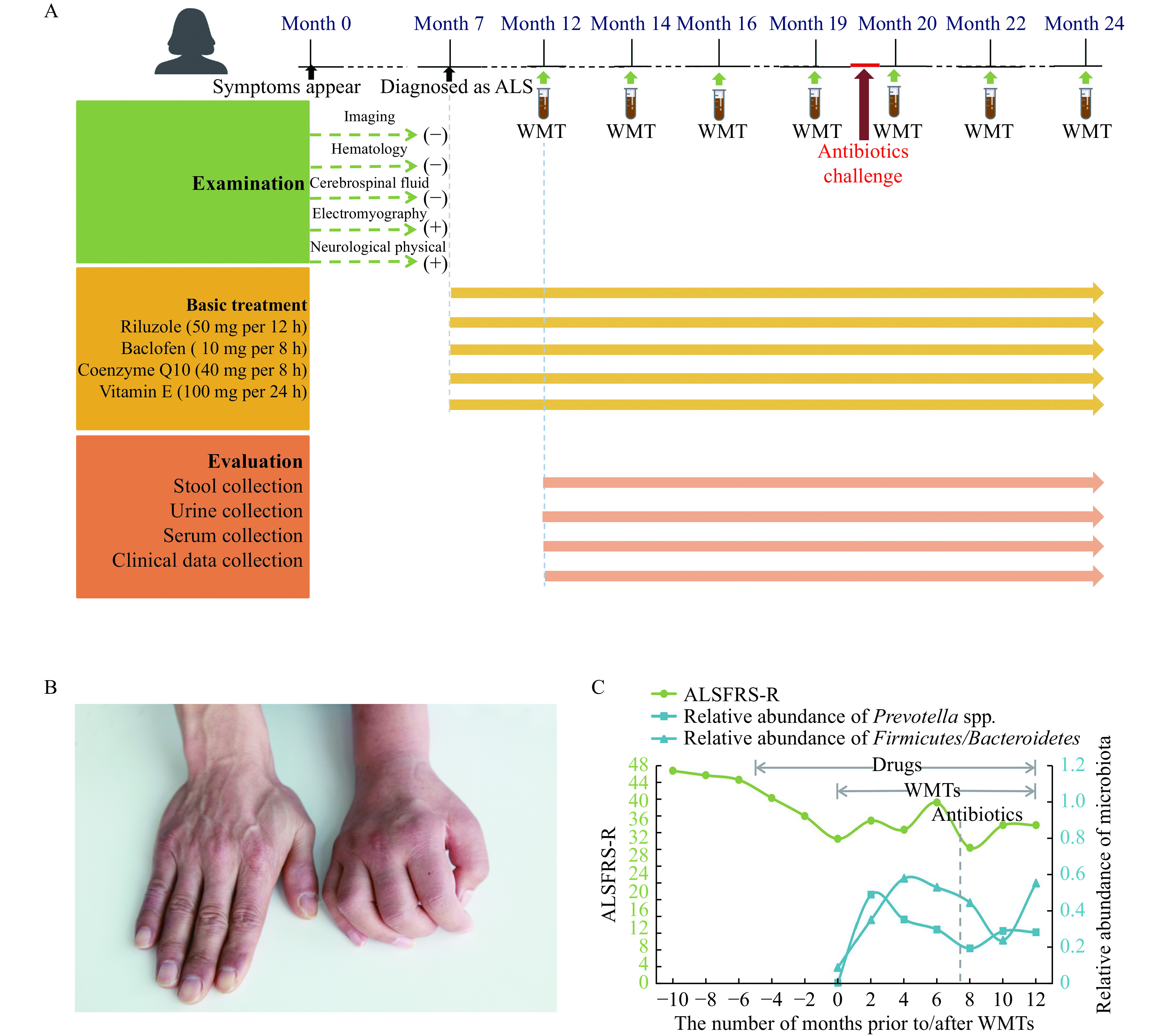
Clinical course, phenotype, and assessment of the patient.

A 48-year-old woman presented with muscle stiffness and weakness in November 2019. She also complained of moderate to severe constipation for the same duration of time. Early in the fall of 2019, she noted stiffening and weakening of her left leg muscles, and later developed muscle fasciculation, followed by amyotrophy, initially involving the left leg, but spreading gradually to the right leg and two arms without obvious sensory symptoms (***Fig. 1***). Investigations including routine biochemistry, hematology, vasculitic screen, metabolic screen, infective screen, respiratory testing and imaging studies of the brain and spine showed nothing abnormal. The patient reported no remarkable history of past illness, and there was no family history of any malignant tumors. Neurologic examination showed muscle atrophy in both legs and increased muscle tone in all extremities with generalized pathological hyperreflexia. Electromyographic and nerve conduction examination revealed denervation in the myotomes innervated by cervical, thoracic, and lumbosacral nerves, supporting the final diagnosis of ALS.

**Figure 2:**
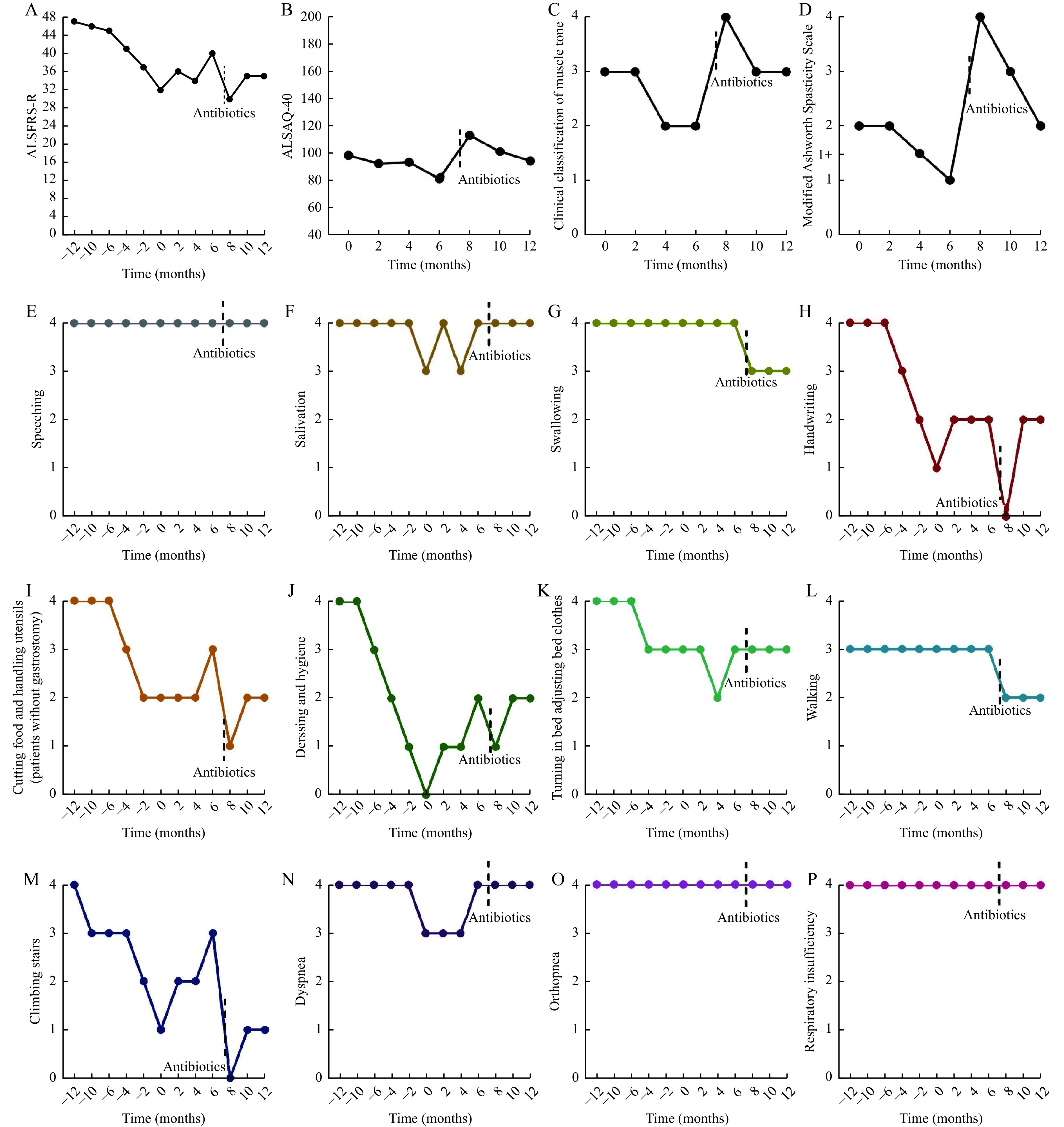
Several clinical assessments of the patient.

She developed aggressive symptoms though she had taken riluzole, baclofen, coenzyme Q10, and vitamin E. In November 2020, she was referred to the Second Affiliated Hospital of Nanjing Medical University for treating complicated refractory constipation with WMT. WMT is an improved methodology of fecal microbiota transplantation (FMT) based on the automatic purification system and washing process^[9]^, whose methodology was standardized by the expert consensus in 2020^[10]^. WMT is a routine clinical technology in China, which only needs ethical approval. We enrolled her for treating constipation and especially evaluated the potential benefits of WMT to ALS. The constipation was well controlled since the first course of fresh WMT through mid-gut tube. Because the satisfactory improvement in the Amyotrophic Lateral Sclerosis Functional Rating Scale-Revised (ALSFRS-R), 40-item Amyotrophic Lateral Sclerosis Assessment Questionnaire (ALSAQ-40), clinical classification of muscle tone and Modified Ashworth Spasticity Scale was observed since the first WMT (***Fig. 1*** and ***Fig 2***), the long-term treatment using WMT through mid-gut or colonic transendoscopic enteral tube was strongly required by the patient and was approved by the expert group. Initiation of WMT brought about a plateau of her symptoms. The impaired balance and gait were gradually improved as well, when muscle tone decreased.

Several months after the initiation of WMT, she suffered an accidental fall that caused scalp trauma, followed by an obvious turning point of ALSFRS-R. The kinds of antibiotics (amoxicillin sodium and clavulanate potassium by injection) were given for 11 days in another hospital to control potential bacterial infections caused by scalp trauma. Her movement limitation reached a peak, and she had to rely on a wheelchair. Her muscle tone returned to her worst status. She was then referred to our department for WMT immediately after her discharge from the previous hospital. This course of the rescue WMT successfully stopped the progression of the disease again with a quick improvement. She could get out of her wheelchair and walk with the help of others, and her muscle tone was also alleviated. The improvement was maintained by the repeated courses of WMT till the submission of this report. All WMTs used freshly washed microbiota, and the dose ranged from 4 to 5 units (one unit = 1.0×10^13^ bacteria) for each WMT. This patient was given a repeated WMT course each two to three months according to Nanjing consensus on the WMT methodology^[9–10]^. The patient did not change or add more drugs for targeting ALS during the follow-up.

**Figure 3:**
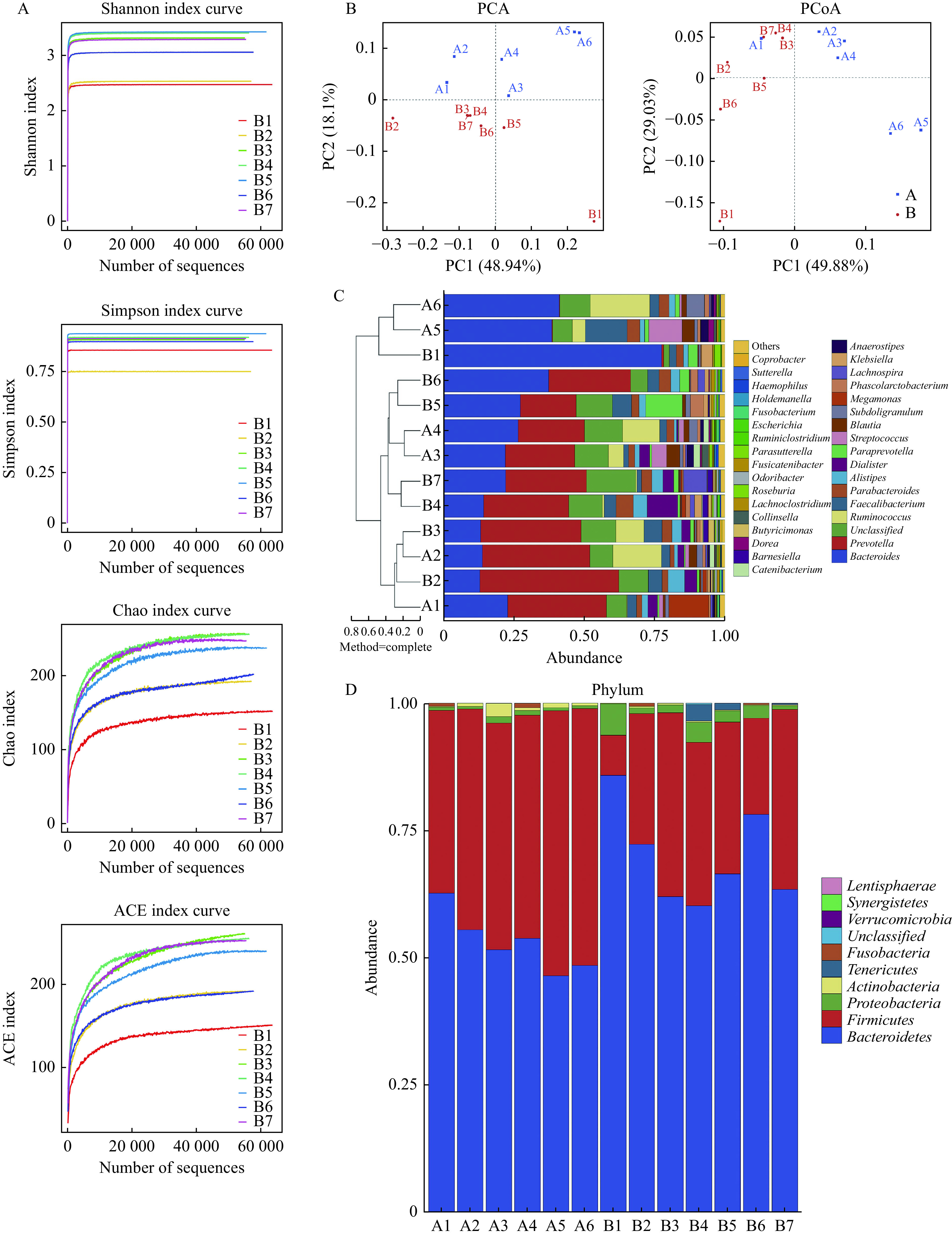
Analysis of fecal microbiota by 16S rRNA sequencing between the patient and donors.

**Figure 4:**
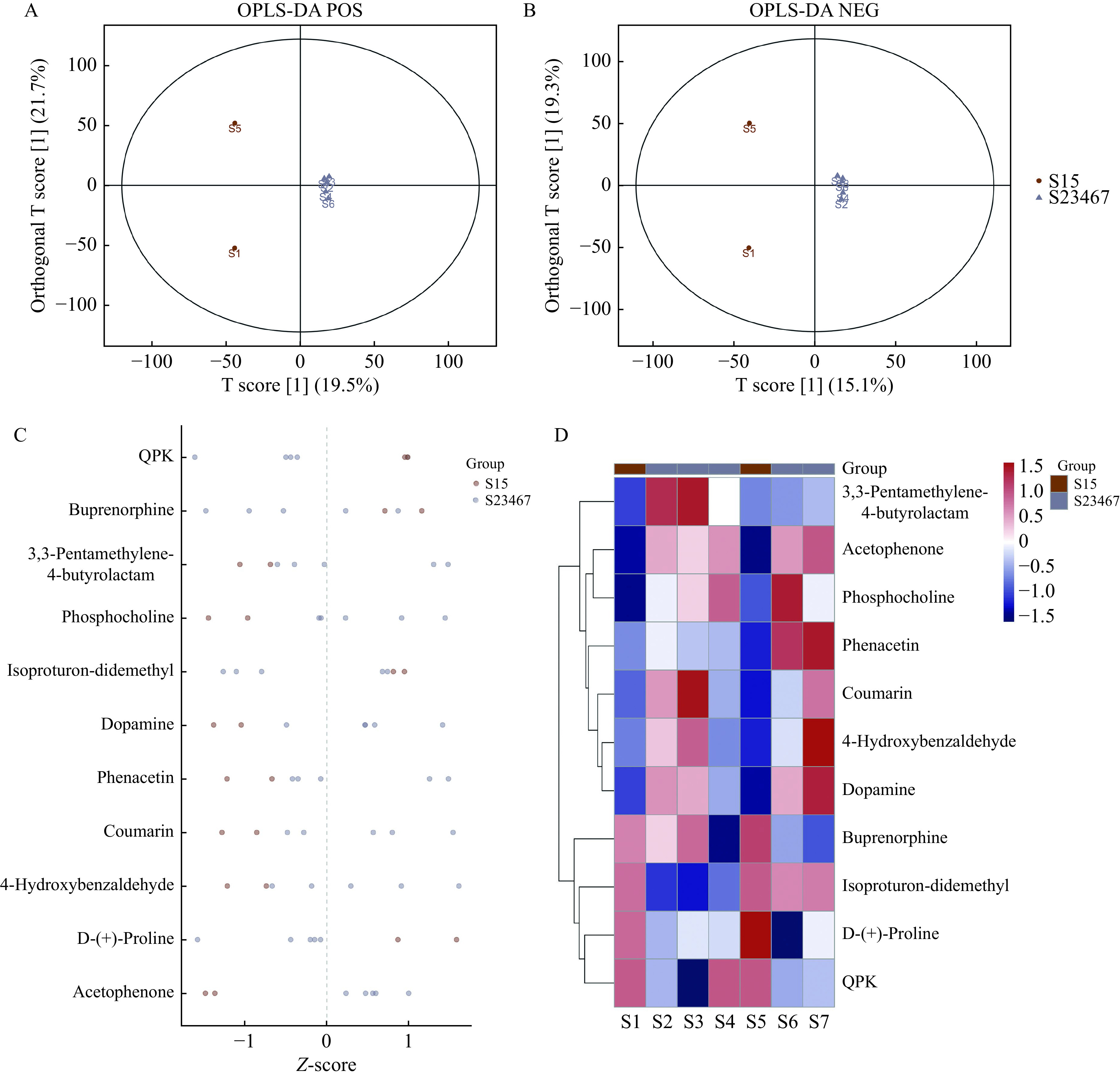
Analysis of serum metabolites by LC-MS before and after WMTs in the patient.

Microbial analysis indicated that the diversity and composition of the gut microbiota from the patient after WMT treatment were closer to those of healthy donors (***Fig. 3***). Some bacteria from the donors colonized successfully after WMTs, such as the *Firmicutes* and *Verrucomicrobia* phylum, *Prevotella* spp. and *Ruminococcus* spp., may contribute to the treatment results of ALS (***Fig. 1***). The changing trend of the potentially harmful bacteria, such as the *Proteobacteria* phylum, changed as the ALSFRS-R after WMTs. Microbiome and metabolome disturbed by antibiotics were reconstructed by WMTs (***Fig. 3*** and ***Fig. 4***). No adverse events were observed within both short-term and long-term follow-up after WMTs.

## Discussion

The concept of microbiota-gut-brain axis has gained a great concern in recent years. However, the very complicated fecal microbiota components determine that the methodology of WMT and the mechanism related to specific microbial matters are different from those of traditional manual FMT. In the present case, we successfully cured her constipation and alleviated her ALS symptoms. Some accidental positive findings from WMT in clinical practice have been reported for bringing benefits to Crohn's disease complicated with epilepsy^[11]^, irritable bowel syndrome with tremor^[12]^, Parkinson's disease with constipation^[13]^, and adult Asperger Syndrome with digestive symptoms^[14]^. WMT from healthy human donors could accelerate the recovery of abnormal changes caused by light-induced stress in Tree Shrews^[15]^. As a note, the terminology of WMT as a specific method of FMT was coined in 2019^[9]^ and released in 2020 by a consensus report^[10]^, washed preparation of fecal microbiota changes the transplantation related safety, quantitative method and delivery^[16]^. Researchers have essentially used the methodology of WMT in clinical practice since 2014, and even replaced FMT in the previous publications.

In this case, some bacteria colonized successfully or accumulated after WMTs. The *Firmicutes* and *Verrucomicrobia* phylum, *Prevotella* spp. and *Ruminococcus* spp. were thought to be donor-derived. The increased *Firmicutes* and decreased *Bacteroidetes* have a beneficial impact on immunity and neuroendocrine of the CNS, while a lower *Firmicutes*/*Bacteroidetes* ratio was used as a marker of intestinal dysbiosis^[17]^. Gioia *et al*^[7]^ and Hertzberg *et al*^[18]^ showed that the gut microbial communities of ALS patients were deficient in *Prevotella* spp. and *Ruminococcus* spp. compared with those of controls^[16,19]^, which were increased after WMTs in this patient. In contrast, some bacteria with potential neurotoxic or pro-inflammatory activity, such as the *Proteobacteria* phylum, were decreased obviously. A prospective longitudinal study has found that the 6-month probiotic treatment influenced the gut microbial composition in patients with ALS^[7]^. Thus, beneficial microbiota interventions, *e.g.*, probiotics/prebiotics or microbiota transplantation may contribute to the diminishing of the inflammation and treating microbiota-gut-brain disorders.

In this case, her clinical symptoms and scores were improved by WMTs in the early stage. However, the evidence based on this period is not enough to confirm the role of WMT on ALS. Bedlack *et al* reported that over 16% of participants experienced a plateaus and that fewer than 1% ever experienced reversals within the Pooled Resource Open-Access ALS Clinical Trials (PRO-ACT) database^[20]^. However, van *et al*^[21]^ suggested that the source of above-mentioned data should be cautiously considered, because the time period of 20 years was too long to match the advancing accuracy of diagnosing ALS. The more important confidence in this case is coming from her experience after her condition was worsened by antibiotics treatment for her scalp trauma. Notably, this patient didn't change the usage of any drugs for ALS during the whole 12-month follow-up, nor did she add any other interventions except WMT. The clear finding is that WMT stopped the deterioration of ALS again. The use of antibiotics was observed on worsening disease progression in the SOD1^G93A^ model of ALS^[6,22]^. In general, the microbiota may influence the CNS neuronal health in different ways by directly producing neuroactive metabolites or toxins, or by indirectly modulating the immune system^[16]^. Another hypothesis is that microbiota can affect circulating levels of inflammatory cytokines that may damage the CNS. Blacher *et al*^[6]^ noted that administering some probiotics such as *Akkermansia muciniphila* can attenuate the symptoms of ALS exacerbated by antibiotics in mice by increasing nicotinamide levels in the CNS. Cox *et al*^[22]^ found the microbiota restrains neurodegenerative microglia to slow down the progress of ALS by the production of metabolites or modulating peripheral immune cells that traffic to the CNS.

There are limitations in the present case report. One case might not be enough to provide a solid evidence for supporting clinical practice, even though this case experienced periods without and with antibiotics treatment. The results from this patient is preliminary and observational, and the protocol of using WMT for other ALS patients at least should put the stage of treating the disease and the delivering way as well as the frequency of WMT into careful consideration. For constituting a treatment recommendation, randomized controlled trials are needed to meet more sustained ALS reversals and clarify the value of WMT^[23]^.

In conclusion, we for the first time reported the positive clinical outcomes of using WMT for treating ALS. Although the disease condition of the present case was exacerbated by the use of antibiotics, the administered WMTs successfully reversed the worse status of the disease through a long-term follow-up. The current clinical findings would open a new window on the microbiota-based treatment for the life-threatening ALS.
